# Externally oriented thinking style increases primary health care use in adolescence

**DOI:** 10.1093/eurpub/ckad041

**Published:** 2023-03-28

**Authors:** Virve Kekkonen, Siiri-Liisi Kraav, Jukka Hintikka, Petri Kivimäki, Outi Kaarre, Tommi Tolmunen

**Affiliations:** Department of Adolescent Psychiatry, Kuopio University Hospital, Kuopio, Finland; Faculty of Health Sciences, University of Eastern Finland, Kuopio, Finland; Faculty of Health Sciences, University of Eastern Finland, Kuopio, Finland; Faculty of Social Sciences, University of Eastern Finland, Joensuu, Finland; Department of Psychiatry, Päijät-Häme Central Hospital, Lahti, Finland; Faculty of Medicine and Health Technology, University of Tampere, Tampere, Finland; Department of Adolescent Psychiatry, Kuopio University Hospital, Kuopio, Finland; City of Helsinki, Vuosaari Outpatient Psychiatry Clinic, Helsinki, Finland; Faculty of Health Sciences, University of Eastern Finland, Kuopio, Finland; Forensic Psychiatry Clinic, University of Eastern Finland, Niuvanniemi Hospital, Kuopio, Finland; Department of Adolescent Psychiatry, Kuopio University Hospital, Kuopio, Finland; Faculty of Health Sciences, University of Eastern Finland, Kuopio, Finland

## Abstract

**Background:**

Alexithymia has been related to adult health care use. We investigated the association between alexithymia and the utilization of primary health care services by adolescents and young adults.

**Methods:**

The participants (*n *=* *751, aged 13–18 years) in this 5-year follow-up study were assessed with the 20-item Toronto Alexithymia Scale (TAS-20) and its three subscales, difficulty identifying feelings (DIF), difficulty describing feelings (DDF) and externally oriented thinking (EOT), and the 21-item Beck Depression Inventory (BDI). Primary health care data were gathered from health care centre registers in 2005–10. Generalized linear models and mediation analyses were used.

**Results:**

An increase in the TAS-20 total score correlated with a higher number of primary health care and emergency care visits, but in multivariate general linear models, TAS-20 total scores were no longer significant. Younger age, female gender and an increase in the baseline EOT score are associated with a higher number of both primary health care and emergency room visits. In females, a smaller change in the EOT score from baseline to follow-up was associated with a higher number of primary health care visits. In mediation analyses, EOT had a direct effect on a higher number of primary health care and emergency room visits, whereas the BDI score mediated the incremental effect of DIF and DDF on visit numbers.

**Conclusions:**

The results suggest that an EOT style independently increases health care use by adolescents, whereas the effects of difficulties identifying and describing feelings on health care use are mediated by symptoms of depression.

## Introduction

### Alexithymia

Alexithymia is a personality construct that represents a reduced ability to identify feelings and distinguish between emotional feelings and the bodily sensations of emotional arousal, and to find words to describe feelings to other people, as well as a limited imagination and a concrete, externally oriented way of thinking.^1^ The most widely used method for measuring alexithymia is the self-rated 20-item Toronto Alexithymia Scale (TAS-20), which has three subscales: difficulty identifying feelings (DIF), difficulty describing feelings (DDF) and externally oriented thinking (EOT).^2^^,^^3^ According to previous studies, high levels of EOT, in particular, have been associated with deficits in cognitive^4^ and automatic emotional processing,^5^ while high levels of DIF and DDF have been associated with emotional over-responding, negative affect^4^ and variations in the current mental health state.^6–8^

Levels of alexithymia have been reported as highest among the youngest adolescents,^8–10^ who are in the middle of pubertal, physical and emotional maturational process.^11^ Alexithymia has been related to low interoceptive awareness,^12^^,^^13^ self-awareness of internal experiences^14^ and changes in affect regulation.^4^^,^^5^^,^^15^

### Alexithymia and health problems

Alexithymia was originally discovered among patients suffering from psychosomatic problems,^16^ and over time, alexithymia has been related to various health problems,^17^^,^^18^ as well as psychosomatic and somatoform disorders.^19–21^ It has been discussed whether alexithymia is secondary to somatic or psychiatric illnesses. However, several studies in adults have supported the relative stability of alexithymia, referring to a personality construct, despite medical or psychiatric disorders.^15^ For example, symptoms of depression overlap with alexithymia, especially the TAS-20 subscales DIF and DDF in adults^6^^,^^22^ and DIF in adolescents.^23^^,^^24^ Depression and subclinical symptoms of depression are common in adolescence and associated with increased use of health care services.^25^

### Alexithymia and health care service utilization

In studies on adults, alexithymia has been related to frequent health care use due to somatic symptoms^26–28^ and health care utilization due to both somatic and psychological symptoms.^29^ However, the current literature lacks studies on alexithymia and the utilization of health care services in adolescence in comparison to corresponding studies on adults.

### Aims of the study

Levels of alexithymia change during adolescence,^8–10^ are relatively stable in adulthood,^15^ and might predict health behaviour and health care utilization.^26–29^ Our aims were to investigate whether (i) the TAS-20 or its subscales are associated with frequent or substantial primary health care and emergency care utilization in adolescence and (ii) the association between the rate of utilization of health care services and alexithymia might be mediated by symptoms of depression.

## Methods

### Participants

The participants were from a 5-year follow-up study of adolescents aged 13–18 years attending comprehensive, upper secondary and vocational schools in Kuopio, which is a city in Eastern Finland currently having approximately 121 000 inhabitants. Two special schools were excluded from the study on the recommendation of their headmasters, as the questionnaires were considered to be too complicated for their developmentally impaired students. Furthermore, those participants not registered as living in Kuopio were also excluded. The baseline data were collected using structured self-rating questionnaires that the participants completed during class periods at school. Methodological aspects of the baseline study setting have previously been described in detail.^30^ The current study used data from the follow-up study on this population.

The original target population comprised 6421 adolescents aged from 11 to 21 years in 2004–5. The response rate was 65.5%, leading to a sample of 4214 adolescents. Girls responded significantly more often compared to boys. Altogether, 43 participants were excluded due to an age of 12 or younger or 19 or older, leading to a final sample of 4171 adolescents. From this population, 1827 (43.8%) provided their consent to be contacted for a follow-up study. A younger age, female gender and a higher number of hobbies were associated with consenting to be contacted.^31^

The follow-up data were collected by mail. The addresses of 1585 participants were retrieved for recruitment by mail (86.8% of those who consented). Finally, 797 (females 70.9%) participated in the follow-up 5 years later (43.6% of those who consented and 50.3% of those whose postal address could be retrieved). Two participants were excluded as outliers due to an age of less than 13 or more than 18 at baseline. On follow-up, the participants were aged 18–23 years (*N* = 795).

### Primary health care outpatient register

Data on the number of primary health care visits, including the utilization of emergency room services, were obtained from Kuopio primary health care registers from 2005 to 2010 and comprised all visits. Data on visits to the primary health care outpatient unit were collected retrospectively from the public health care centre of the city of Kuopio. Primary health care comprised general practitioners and nursing services in polyclinics, schools and student health care units, and emergency room services. Emergency room services were located at Kuopio University Hospital and provided in collaboration with local public primary, secondary and tertiary (specialist medical) health care. In this data set, the supply of health care services in the city of Kuopio was equally available for all participants and remained constant throughout the research period. Thus, only differences in the utilization of health care services were examined in this study.

For all visits to the primary health care outpatient unit, data on the number of visits and the visit location were gathered from the medical records. Personal identification numbers were used to match follow-up survey data with health care register data.

### Ethical considerations

All procedures performed in studies involving human participants were in accordance with the ethical standards of the institutional and/or national research committee and with the 1964 Helsinki declaration and its later amendments or comparable ethical standards. The study design was approved by the Research Ethics Committee of Kuopio University Hospital (no. 77/2010) and the Finnish Institute for Health and Welfare (no. THL/1628/5.05.00/2010).

### Measurements

Baseline data were collected with a self-rated questionnaire that included age and gender. The prevalence of alexithymia was assessed at baseline and on follow-up using the Finnish version of the TAS-20.^1^^,^^2^ Studies on Finnish populations have demonstrated the validity of the TAS-20 in adolescents, although no cut-off scores have been established.^9^^,^^19^ The TAS-20 has three subscales: (i) DIF (seven items: 1, 3, 6, 7, 9, 13 and 14), (ii) DDF (five items: 2, 4, 11, 12 and 17) and (iii) EOT (eight items: 5, 8, 10, 15, 16, 18, 19 and 20). The scores for the three TAS-20 subscales were calculated by using the original factor structure of Bagby et al.^2^

Depressive symptoms were assessed with the 21-item Beck Depression Inventory (BDI). The BDI questions target cognition, behaviour, emotions and somatic complaints.^32^^,^^33^ The BDI has been validated for adolescents.^34^

### Statistics

The internal consistency and reliability of the TAS-20 total and subscales were assessed using Cronbach’s alpha. With the poor values for baseline DDF and EOT as exceptions, Cronbach’s alpha values indicated acceptable internal consistency: TAS-20 total score 0.77, DDF 0.32, DIF 0.82 and EOT 0.48 at baseline, and TAS-20 total score 0.83, DDF 0.79, DIF 0.83 and EOT 0.65 on follow-up.

First, the characteristics of the study sample were investigated. BDI scores at baseline were non-normally distributed, whereas all other continuous variables (age, TAS-20 total, TAS-20 subscale scores and changes in TAS-20 scores) were normally distributed. The total number of primary health care and emergency care visits had a peak for zero values, but after natural logarithmic transformation, these continuous variables were normally distributed. The chi-squared test was used to analyse the group differences in the categorical variables (gender). The Student’s *t*-test was used in the comparisons of normally distributed variables (age, alexithymia and alexithymia subscales) and the non-parametric Mann–Whitney U-test in the comparisons of continuous variables with a non-normal distribution (BDI). Then, correlations between continuous variables were investigated. The associations between continuous variables were measured with Spearman’s rho (ρ) values.

Next, the number of primary health care and emergency room visits during the 5-year follow-up and associated factors were investigated with generalized linear models with a negative binomial distribution and logarithm link function due to the high number of zero values in the data, indicating no visits, and the non-normal distribution of some independent values, as described above. The numbers of primary health care and emergency room visits were used as dependent variables and all other variables, such as age, gender, TAS-20 total or TAS-20 subscale scores at baseline, the changes in TAS-20 total or TAS-20 subscale scores from baseline to follow-up and the BDI score at baseline, were used as independent variables. Analyses were performed first with the TAS-20 total score and then with TAS-20 subscale scores, as well as all other variables in the same model.

Finally, to further examine the relationship between alexithymia and the use of health care services, including emergency room visits, we determined whether depressive symptoms represented by the BDI score mediated the connection between the subscales of alexithymia and health care service utilization. In these analyses, logarithmic transformations of the health care services utilization variables were used. The connections between TAS-20 subscales (DIF, DDF and EOT) and health service utilization were assessed using the PROCESS macro (v. 3.5.3) for SPSS.^35^ We used a simple mediation model (model 4) and inserted the TAS-20 subscales into the analysis one at a time. For mediation analyses, 5000 percentile bootstrap samples were used.

*P*-values below 0.05 were considered to indicate statistical significance. All the models were tested for multicollinearity, and all variance inflation factors (VIF) were less than 5. All analyses were conducted with IBM SPSS (version 27) statistical software.

## Results

### Characteristics of the study sample

Altogether, there were 793 participants, from whom primary health care data could be retrieved for 441 participants [55.6%; 303 (68.7%) females] and emergency room services visit data for 287 participants (36.2%; 71.8% females) during 2005–10. The total number of visits was 15 348, including 1641 emergency room visits. Altogether, 751 participants had both baseline and follow-up data on alexithymia, and of these, 326 participants attended a total of 12 793 primary health care visits.

In correlation analyses, younger age and an increase in baseline TAS-20 total, EOT and BDI scores were associated with both total primary health care visits and total emergency room visits. Among females but not males, an association was detected between TAS-20 total scores and total health care visits, and between BDI scores and total health care and emergency room visits. The correlations between TAS-20 total and subscale scores and the BDI score at baseline are presented in table 1.

**Table 1:** Spearman’s rho (ρ) between primary health care and emergency room visits, and participants’ age, Beck Depression Inventory (BDI) scores and the 20-item Toronto Alexithymia Scale (TAS-20) total and subscale scores at baseline

All participants		Age	BDI	TAS	DIF	DDF	EOT
Number of all primary health care services visits in 2005–10	Correlation coefficient	−0.268^**^	0.097^**^	0.111^**^	0.025	0.039	0.152^**^
	*P*	<0.001	0.007	0.002	0.482	0.280	<0.001
Emergency room visits in 2005–10	Correlation coefficient	−0.143^**^	0.093^**^	0.080^*^	0.023	0.029	0.106^**^
	*P*	<0.001	0.009	0.028	0.528	0.425	0.003

Males		Age	BDI	Total	DIF	DDF	EOT

Number of all primary health care services visits in 2005–10	Correlation coefficient	−0.352^**^	0.052	0.101	−0.063	0.003	0.269^**^
	*P*	<0.001	0.428	0.132	0.343	0.961	<0.001
Emergency room visits in 2005–10	Correlation coefficient	−0.104	0.085	0.029	−0.057	−0.020	0.170^*^
	*P*	0.112	0.198	0.659	0.389	0.759	0.011

Females		Age	BDI	Total	DIF	DDF	EOT

Number of all primary health care services visits in 2005–10	Correlation coefficient	−0.243^**^	0.105^*^	0.113^**^	0.048	0.047	0.140^**^
	*P*	<0.001	0.014	0.009	0.266	0.280	0.001
Emergency room visits in 2005–10	Correlation coefficient	−0.158^**^	0.096^*^	0.095^*^	0.048	0.044	0.100^*^
	*P*	<0.001	0.024	0.028	0.266	0.307	0.019

BDI, Beck Depression Inventory; TAS, TAS-20 total scores; DIF, difficulty identifying feelings; DDF, difficulty describing feelings; EOT, externally oriented thinking. Significant *p*-values: *<0.050, **<0.010, ***<0.001.

### Results from generalized linear model analyses

Alexithymia total scores and changes in total scores between baseline and follow-up were not associated with the rate of utilization of primary health care services or emergency services when adjusted for age, gender and the BDI score at baseline.

Younger age, female gender, an increase in the baseline EOT score and an increase in the baseline BDI score are associated with a higher number of primary health care visits. In females, an increase in the DIF score and a decrease in the EOT score from baseline to follow-up were associated with a higher number of primary health care visits. Additionally, female gender and an increase in the baseline EOT score are associated with a higher number of emergency room visits. In females, younger age and an increase in the baseline BDI score are associated with a higher number of emergency room visits. Changes in TAS-20 subscale scores from baseline to follow-up were not associated with the number of emergency room visits (table 2).

**Table 2:** Generalized linear model analysis of all visits to primary health care services, emergency room visits and the 20-item Toronto Alexithymia Scale (TAS-20) subscale scores at baseline, subscale scores between baseline and follow-up and Beck Depression Inventory (BDI) scores at baseline

	Variable	All			Males			Females		
		*B*	S.E.	*P*	*B*	S.E.	*P*	*B*	S.E.	*P*
All primary health care services visits	Gender, male	−0.71	0.09	<0.001						
	Age, years	−0.22	0.03	<0.001	−0.28	0.05	<0.001	−0.20	0.03	<0.001
	DIF at baseline	−0.02	0.01	0.131	−0.02	0.02	0.371	−0.01	0.01	0.394
	DDF at baseline	−0.01	0.01	0.665	−0.01	0.03	0.615	−0.01	0.02	0.681
	EOT at baseline	0.05	0.01	<0.001	0.07	0.02	<0.001	0.04	0.01	0.007
	BDI at baseline	0.04	0.01	<0.001	0.04	0.02	0.013	0.04	0.01	<0.001
	Gender, male	−0.56	0.09	<0.001						
	Age, years	−0.27	0.03	<0.001	−0.33	0.05	<0.001	−0.26	0.03	<0.001
	DIF change	0.01	0.01	0.231	−0.02	0.02	0.324	0.02	0.01	0.045
	DDF change	0.01	0.01	0.430	−0.02	0.02	0.286	0.01	0.01	0.316
	EOT change	−0.02	0.01	0.056	0.02	0.02	0.172	−0.03	0.01	0.005
	BDI at baseline	0.03	0.01	<0.001	0.04	0.01	0.016	0.02	0.01	0.001
Emergency room visits	Gender, male	−0.52	0.11	<0.001						
	Age, years	−0.10	0.03	0.003	−0.08	0.06	0.191	−0.10	0.04	0.006
	DIF at baseline	−0.02	0.01	0.091	−0.01	0.02	0.620	−0.03	0.02	0.080
	DDF at baseline	0.00	0.02	0.825	0.01	0.03	0.866	0.00	0.02	0.836
	EOT at baseline	0.05	0.01	<0.001	0.06	0.02	0.003	0.04	0.02	0.010
	BDI at baseline	0.04	0.01	<0.001	0.02	0.02	0.434	0.04	0.01	<0.001
	Gender, male	−0.41	0.10	<0.001						
	Age, years	−0.14	0.03	<0.001	−0.12	0.07	0.060	−0.15	0.04	<0.001
	DIF change	0.00	0.01	0.955	−0.02	0.02	0.435	0.01	0.01	0.622
	DDF change	0.01	0.01	0.414	0.00	0.03	0.917	0.01	0.02	0.409
	EOT change	0.00	0.01	0.776	0.02	0.02	0.302	−0.01	0.01	0.384
	BDI at baseline	0.03	0.01	<0.001	0.02	0.02	0.337	0.03	0.01	0.001

BDI, Beck Depression Inventory; DIF, difficulty identifying feelings; DDF, difficulty describing feelings; EOT, externally oriented thinking.

### Results from mediation analysis

**Figure 1:**
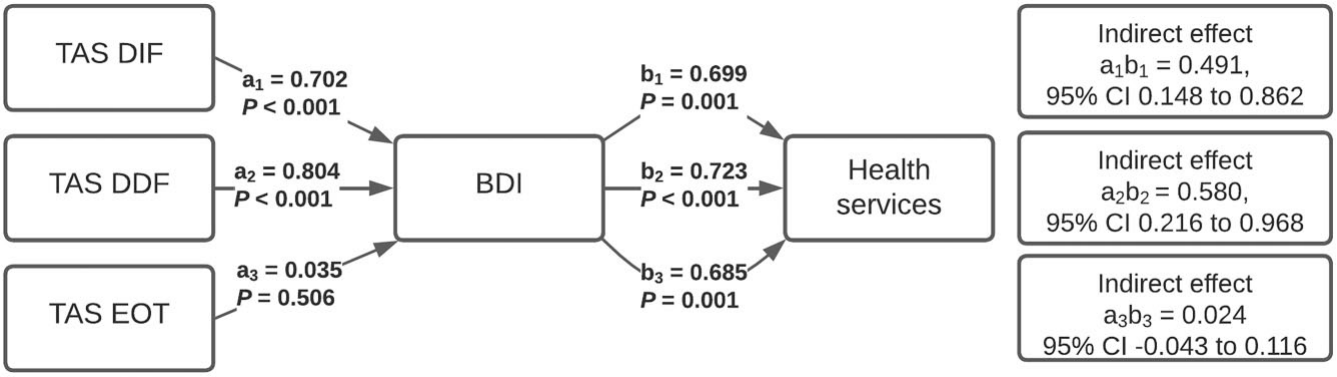
Indirect effects of the 20-item Toronto Alexithymia Scale (TAS-20) subscales difficulty identifying feelings (TAS DIF), difficulty describing feelings (TAS DDF) and externally oriented thinking (TAS EOT) on health service use, mediated by depressive symptoms. All the subscales were inserted into the simple mediation model one at a time

**Figure 2:**
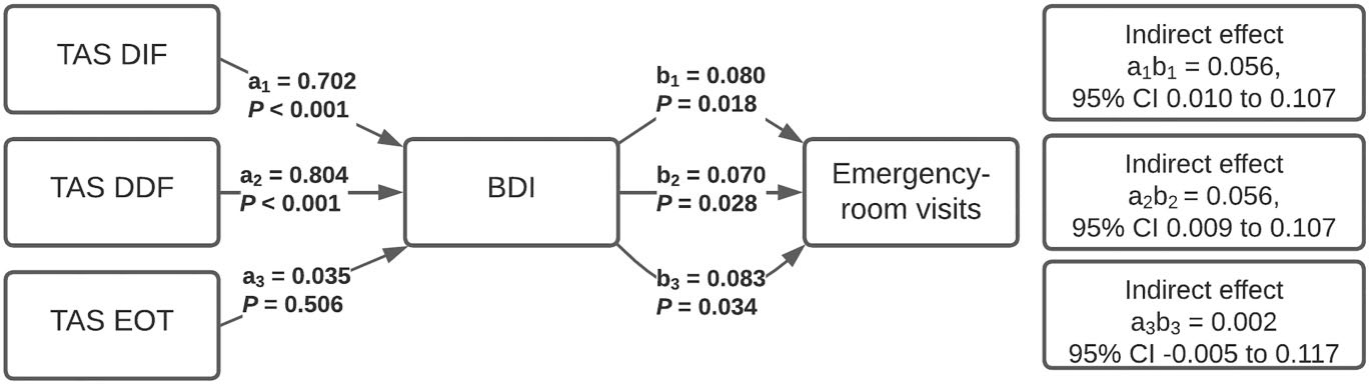
Indirect effects of the 20-item Toronto Alexithymia Scale (TAS-20) subscales difficulty identifying feelings (TAS DIF), difficulty describing feelings (TAS DDF) and externally oriented thinking (TAS EOT) on emergency room visits, mediated by depressive symptoms. All the subscales were inserted into the simple mediation model one at a time

According to simple mediation analyses, BDI scores, indicating depressive symptoms, mediated the effects that DIF and DDF scores had on the number of primary health care and emergency room visits (figures 1 and 2, respectively). When BDI scores were inserted into the mediation models as mediators, the direct effects between DIF, DDF and primary health care and emergency room visit counts were no longer statistically significant. EOT had a direct incremental effect on the number of primary health care and emergency room visits.

## Discussion

### Main results

This research sought to address whether (i) the TAS-20 or its subscales are associated with frequent or substantial primary health care and emergency care utilization in adolescence and (ii) the association between the utilization of health care services and alexithymia is mediated by depressive symptoms. We found that an increase in the TAS-20 total score correlated with a higher number of primary health care and emergency care visits, and an increase in the baseline EOT score was associated with a higher number of both primary health care and emergency room visits. In females, a smaller change in the EOT score from baseline to follow-up was associated with a higher number of primary health care visits. In mediation analyses, EOT had a direct effect on a higher number of primary health care and emergency room visits, whereas the BDI score mediated the incremental effect of DIF and DDF on visit numbers.

### Comparison with previous literature

In correlation analyses, the baseline alexithymia total score and EOT score are associated with an increased number of health care visits. Furthermore, when adjusted for age, gender and symptoms of depression in general linear models, alexithymia total scores did not associate with the primary health care or emergency room visit number. An association between alexithymia and the number of visits to health care services has been reported among adults.^26–29^ Furthermore, in adults, an association between frequent primary health care use and alexithymia has been related to psychological distress among both genders.^29^ To the best of our knowledge, previous studies on adolescent alexithymia and primary health care use were not found.

When alexithymia subscales were investigated concurrently in general linear models, an increase in the baseline EOT score was associated with higher numbers of primary health care and emergency room visits in both genders. Furthermore, in females, a smaller absolute change in EOT scores from baseline to follow-up was associated with a higher number of primary health care visits. In a 1-year follow-up study, baseline EOT was independently linked to a decreased use of outpatient treatment, i.e. to not undergoing psychotherapy, in young adult college students.^28^ Among adult patients with chronic anxiety symptoms (*n *=* *312), EOT scores were higher among those who never received professional treatment compared to those with current or previous treatment.^36^ These findings from previous studies on adults are contrary to our findings in adolescents. In adult somatoform disorder patients (*n *=* *196), higher EOT was associated with less confrontive coping strategies in treatment, possibly due to difficulties in observing the source of stress and a lack of confidence in problem solving.^37^ These findings are in line with observations that alexithymia is common among patients with psychosomatic problems,^16^ and that it might emerge as difficulties in interpreting internal experiences.^14^ As far as we are aware, no previous studies have examined the association between EOT and health care utilization among adolescents.

Our second aim was to investigate whether the association between the utilization of health care services and alexithymia is mediated by symptoms of depression measured with the BDI score. An increase in the BDI score from baseline to follow-up is associated with a higher number of primary health care visits in both genders, and with a higher number of emergency room visits in females. Mediation analyses revealed that depressive symptoms mediated the relationship between DIF and DDF scores and the number of both health care service and emergency room visits. More interestingly, when depressive symptoms were inserted as a mediator into the mediation models, the direct relationships between DIF and DDF and the number of health care service and emergency room visits were no longer statistically significant. However, EOT had a direct relationship with a higher number of both health service and emergency room visits, and these connections were not mediated by depressive symptoms. It has been debated whether alexithymia is secondary to psychiatric disorders or rather a stable personality trait independent of psychiatric disorders.^15^ Depression is a disorder of impaired emotion regulation that is common in adolescence^25^ and may overlap with alexithymia, especially the TAS-20 subscales DIF and DDF.^6^^,^^22–24^ In our study, we found that depressive symptoms functioned as a mediator between both DIF and DDF and health service use, as well as emergency room visits. In the case of EOT, the mediating effect of depressive symptoms was not significant. Our study is in line with previous studies suggesting an overlap between depression and difficulty identifying and describing feelings, but not between depression and EOT.

The youngest adolescents have the highest levels of alexithymia,^8–10^ which might lower their ability to identify internal experiences^14^ and to regulate emotions.^4^^,^^5^ Therefore, a low ability to become aware of and to recognize one’s internal experiences^12^^,^^13^ might be the reason for the somatic manifestation of various psychological symptoms in adolescents.^11^ Consequently, recurrent health care visits in adolescence might refer to unrecognized mental health symptoms and the need for care. However, in the future, the relationship between an EOT style and health care utilization by adolescents requires further investigation.

### Strengths and limitations

The main strength of this study was its longitudinal setting, examining a large variety of possible confounding variables, combined with the use of retrospective medical health care registers. The high study dropout rate and female over-representation can be considered as limitations.^31^ Only differences in the utilization of health care services were examined in this study. Thus, possible differences between years in each participant’s visit number, as well as differences in the availability of health care services, might have caused differences in utilization, resulting in bias. However, the participants were approximately of the same age and lived in the same area, and the risk of bias caused by the availability of services is thus likely to have been small. Despite the modern medical record systems, documentation of the reasons for health care visits was incomplete. Possible differences between employees in their way of reporting medical examinations in patient files may have caused some systematic bias. The psychometric properties of the TAS-20 have been satisfactory among young adults.^15^ However, due to the development of affect regulation in adolescence, the TAS-20 may have shortcomings among these individuals, especially in early adolescence (ages 12–14), due to their still developing affect regulation.^15^^,^^38^ Utilizing structured clinical interviews to obtain diagnoses would have been a more exact means to evaluate the mental health status than self-reported questionnaires. However, due to the large sample size and limited resources, we were unable to utilize these types of tools with this dataset. Self-reporting of health may be vulnerable to bias, e.g. due to embarrassment. According to our previous study on sample selection biases, more frequent study participation was associated with female gender, a higher number of hobbies and better school performance, as well as symptoms of depression and anxiety.^31^

### Summary and conclusions

EOT at baseline is associated with a higher number of both primary health care and emergency room visits. Symptoms of depression are associated with a higher number of primary health care visits in both genders, and with a higher number of emergency room visits in females. In mediation analyses, symptoms of depression mediated the effect that DIF and DDF had on both primary health care and emergency room visit numbers. Our results suggest that EOT may independently affect adolescent health care utilization, whereas difficulties identifying and describing feelings may affect adolescent health care utilization via symptoms of depression.

## Data Availability

The data on which the findings of this study have been based are available from the corresponding author upon reasonable request. The association between alexithymia and adolescent health care use was investigated. Depressive symptoms mediated the effect of alexithymia on health care use. An externally oriented thinking style might influence health care use by adolescents.
